# Steroids in the Treatment of IgA Nephropathy to the Improvement of Renal Survival: A Systematic Review and Meta-Analysis

**DOI:** 10.1371/journal.pone.0018788

**Published:** 2011-04-12

**Authors:** Yu-Hao Zhou, Li-Gong Tang, Shi-Lei Guo, Zhi-Chao Jin, Mei-Jing Wu, Jia-Jie Zang, Jin-Fang Xu, Chun-Fang Wu, Ying-Yi Qin, Qing Cai, Qing-Bin Gao, Shan-Shan Zhang, Dand-Hui Yu, Jia He

**Affiliations:** 1 Department of Health Statistics, Second Military Medical University, Shanghai, China; 2 Department of Anatomy, Second Military University, Shanghai, China; 3 Department of Rheumatology and Immunology, Changhai Hospital, Second Military Medical University, Shanghai, China; 4 Academic Journal of Second Military Medical University, Shanghai, China; 5 Department of Urology, Wuhan General Hospital, Guangzhou Command PLA, Wuhan, China; 6 Tumor Immunology and Gene Therapy Center, Eastern Hepatobiliary Surgery Hospital, Second Military Medical University, Shanghai, China; INSERM, France

## Abstract

**Background:**

Studies have shown that steroids can improve kidney survival and decrease the risk of proteinuria in patients with Immunoglobulin A nephropathy, but the overall benefit of steroids in the treatment of Immunoglobulin A nephropathy remains controversial. The aim of this study was to evaluate the benefits and risks of steroids for renal survival in adults with Immunoglobulin A nephropathy.

**Methodology and Principal Findings:**

We searched the Cochrane Renal Group Specialized Register, Cochrane Controlled Trial Registry, MEDLINE and EMBASE databases. All eligible studies were measuring at least one of the following outcomes: end-stage renal failure, doubling of serum creatinine and urinary protein excretion. Fifteen relevant trials (n = 1542) that met our inclusion criteria were identified. In a pooled analysis, steroid therapy was associated with statistically significant reduction of the risk in end-stage renal failure (RR: 0.46, 95% CI: 0.27 to 0.79), doubling of serum creatinine (RR = 0.34, 95%CI = 0.15 to 0.77) and reduced urinary protein excretion (MD = −0.47g/day, 95%CI = −0.64 to −0.31).

**Conclusions/Significance:**

We identified that steroid therapy was associated with a decrease of proteinuria and with a statistically significant reduction of the risk in end-stage renal failure. Moreover, subgroup analysis also suggested that long-term steroid therapy had a higher efficiency than standard and short term therapy.

## Introduction

Immunoglobulin A (IgA) nephropathy or Berger's disease is the most common form of primary glomerulonephritis worldwide [1]–[5]. It is one of the major factors leading to end stage renal failure. About 15–20% of patients with apparent onset IgA nephropathy will develop end stage renal failure within 10 years, and 30–40% within 20 years [6]–[8]. The disease is characterized by accumulation of polymeric IgA1-containing complexes in the mesangial areas [9], [10]. IgA nephropathy can occur at all ages, but most commonly in the second and third decades of life, with a male gender preference [11]. Episodic macroscopic hematuria has been reported as the most common clinical manifestation (40–50% of cases) of IgA nephropathy patients, especially in the second and third decades of life [12]. It has also been found that <5% cases were complicated by upper respiratory infection or acute kidney injury (AKI), the most often in the elderly [13], [14].

Only in recent years has the pathogenetic mechanism underlying IgA nephropathy been found, which can be divided into three essential steps: i) generation of abnormal IgA1 and formation of IgA1 complexes; ii) generation of mesangial injury mediated by interaction of IgA1 complexes with mesangial IgA receptors, and iii) progression of IgA-mediated mesangial injury towards renal failure [15], [16]. Moreover, IgA nephropathy is highly variable both clinically and pathologically [11], [16]. The clinical features ranged from asymptomatic hematuria to rapid progressive glomerulonephritis (RPGN) [14]. IgA nephropathy is most often associated with microscopic hematuria or recurrent macroscopic hematuria, and spontaneously resolving acute renal failure can also occur; this condition can sometimes lead to chronic kidney disease as well [13], [17]. Pathologically, a spectrum of glomerular lesions can be seen, and mesangial proliferation with prominent IgA deposition can be observed in almost all biopsies [10], [18].

The goal of treatment for IgA nephropathy is to prevent sequelae. At the beginning of 1980s, corticosteroid treatment was firstly proposed for paediatric patients with IgAN and nephritic syndrome, which was generally seen as predictive of progressive renal insufficiency [16]. However, recent Cochrane systematic review failed to show the benefit of such treatment for adults, which only demonstrated the benefits and risks of steroids in renal survival in children with IgA nephropathy [19]. Another important review provided an unclear conclusion because it included trials with low quality [20]. Several clinical trials have been completed recently [21], [22], which demonstrated both the benefits and risks of steroids in renal survival in adults with IgA nephropathy.

We performed a systematic review and meta-analysis including the most updated evidence in the effects of steroid therapy on end-stage renal failure, doubling of serum creatinine, urinary protein excretion, and possible side-effects in patients with IgA nephropathy.

## Methods

### Search Strategy

We systematically searched the English literature to identify all relevant, randomized, double-blind, placebo-controlled trials regardless of publication status (published, unpublished, in press, and in progress), and to examine the effects of steroids on IgA nephropathy. Relevant trials were identified with the following procedure:

Electronic Searches: We searched the electronic databases MEDLINE, EMBASE, Cochrane Renal Group Specialized Register and Cochrane Controlled Trial Registry for relevant trials to a time limit of Apr. 10, 2010 using “Steroids” and “IgA nephropathy” as the search terms.

Other sources: We contacted authors to obtain any possible additional published or unpublished data and we searched the proceedings of the annual meeting in the Cochrane Renal Group Specialized Register. In addition, we searched for ongoing RCTs in the metaRegister of controlled trials using the search terms “Steroids” and “IgA nephropathy” as the above statement.

### Study Selection

**Figure 1:**
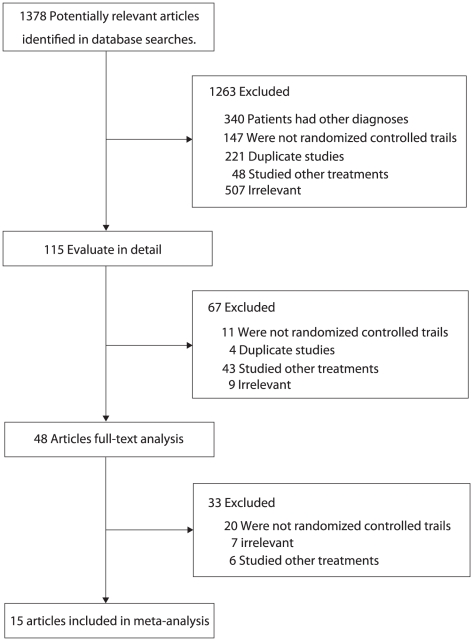
Flow diagram showing the number of citations identified, retrieved and included in final analysis.

Two authors (Shi-Lei. Guo, Chun-Fang. Wu) screened the primary searching results to identify any relevant trials that required further retrieval (full text or abstract), and then they independently reviewed these studies, looking for trials that met the inclusion criteria (Figure 1). The inclusion criteria were broad so that it would be possible to include all evidence now existing, regardless of the study language, study quality, sample size and other factors. The included studies were randomized controlled trials or quasi-randomized controlled trials and any type of treatment with steroids (methylprednisolone or prednisolone) was compared to treatments without steroids.

### Data extraction

Data extraction was performed by two reviewers (Shi-Lei. Guo, Chun-Fang. Wu) independently using a standardized extraction form. If there was any disagreement between the two reviewers, it would be settled by discussion with a third reviewer (Yu-Hao. Zhou) until a consensus was reached.

Data were extracted from the included trials in terms of patients' characteristic, doses and modalities of treatment, methodological characteristics of the trials. The primary reported outcomes were summarized in tables. One author (Yu-Hao Zhou) entered the data into computer, and three other authors (Jia-Jie. Zang, Chun-Fang. Wu, and Jin-Fang. Xu) checked it.

### Assessment of Methodological Quality

The quality of the trials was assessed according to the pre-fixed, including allocation concealment, blinding, Intention-to-treat analysis, and completeness of follow-up. Judgments regarding the presence of methodological biases were made according to Cochrane criteria guidelines [23]. Any disagreement among the three authors (Shi-Lei. Guo, Chun-Fang. Wu, and Jin-Fang. Xu) was settled by discussion with a fourth author (Yu-Hao. Zhou) until a consensus was reached.

### Data Analysis

The effect of steroids on renal survival was evaluated in terms of incidence of end stage renal failure, doubling of serum creatinine and urinary protein excretion [24]–[26]. We used relative risk (RR) with 95% confidence intervals (CIs) for binary data and mean difference (MD) with 95% confidence intervals (CIs) for continuous data. We also did subgroup analysis to assess potential effect change for all results based on the type of control drug and duration of treatment. All estimates of effect were derived using a random-effects model with Mantel-Haenszel Statistics. Heterogeneity of treatment effects between studies was investigated visually by scatter plot and statistically by the heterogeneity
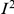
statistic. When the
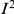
statistic was 0% to 40%, it indicates that heterogeneity is unimportant, 30% to 60% indicates moderate, 50% to 90% indicates substantial and 75% to 100% indicates considerable heterogeneity [27]. The P value is from the 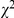 test. All the reported *P* values are two-sided and values of *P*<0.05 was regarded as statistically significant for all included studies. All analyses were calculated using STATA (version 10.0).

## Results

We identified 1,378 articles from our initial electronic search, of which 1,263 were excluded during an initial review (title and abstract), we retrieved the full text for the remained 115 articles, and 15 clinical trials met the inclusion criteria (Figure 1): they contributed to the study of 1,542 patients with IgA nephropathy in this systematic review and meta-analysis. Table 1 summarized the characteristic of these studies and the important baseline information of the included patients. The follow-up for patients ranged from 3 to 281 months, and the number of patients included in every study ranged from 20 to 702. Of these studies, two trials [28], [31] compared steroids with anti-platelet drugs. Five trials [29], [32], [33], [35], [37] compared steroids with anti-inflammatory drugs and/or anti-thromboyte. Six trials [21], [22], [30], [34], [39], [40] compared steroids with supportive treatment. Placebo was compared with steroids in one trial [36] and only one trial [38] gave no treatment for the control group. In addition, three trials [34], [36], [38] investigated short-term steroid therapy, five trials [21], [22], [30], [32], [40] investigated standard-term steroid therapy and remaining seven trials [28], [29], [31], [33], [35], [37], [39] investigated long-term steroid therapy. Some of the included studies keep low-quality because many trials have design limitations. Table 2 shows the detailed quality of included individual trials.

**Table 1:** Characteristic of the included studies.

Source	Population	No. of Patients	Interventions	Baseline renal function and/or proteinuria	Follow up (month)
Katafuchi R 2008 [28]	Been biopsied with primary IgA nephropathy	702	(1) Methylprednisolone 1 g/day 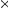 3 days plus Prednisolone 30 mg/day 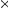 2 years(2) Prednisolone 60 mg/day 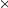 4 weeks and then tapered off within 1 year, Prednisolone 20 mg or 30 mg/day 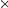 1 month and Prednisolone was tapered and maintained for at least 2 years(3) Anti-platelet agent or with no medication	Serum creatinine(0.98 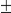 0.58 mg/dl)	62
Koike M 2008[29]	Light microscopic findings	48	(1) Prednisolone was 0.4 mg/kgBW/day (20–30 mg/day) 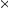 4 weeks, then reduced to 10–20 mg on alternate days 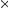 12 months and 5–10 mg on alternate days for a subsequent year(2) dipyridamole or zilazep hydrochloride 150 or 300 mg/day	Steroid group serum creatinine was 0.92 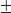 0.26 mg/dl and non-steroid group was 1.15 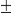 0.35 mg/dl	24
Pozzi C 2004[30]	Biopsy-proven Ig A nephropathy	86	(1) Methylprednisolone 1 g/day 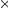 3 days and again 2 and 4 months later, other day oral prednisolone 0.5 mg/kg 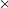 6 months(2) Supportive treatment	Urinary protein excretion levels of 1–3.5 g/d	120
Moriyama T 2004[31]	Been diagnosed based on light-microscopic findings	60	(1) Prednisolone 0.8 mg/kg/day 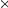 4 weeks, then reduced to 20–30 mg on alternate days 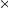 12 months and 20 mg on alternate days 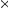 12 months(2) Anti-platelet agent	Steroid group serum creatinine was 1.21 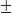 0.44 mg/dl and non-steroid group was 1.27 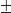 0.33 mg/dl	54.6
Katafuchi R 2003[32]	Biopsy-proven new Ig A nephropathy	103	(1) Prednisolone 20 mg/day 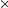 1 month, 15 mg/day 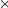 1 month, 10 mg/day 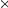 1 month, 7.5 mg/day 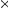 3 months and 5 mg/day 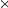 18 months(2) dipyridamole 150 or 300 mg/day	Steroid group serum creatinine was 0.92 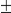 0.24 mg/dl and non-steroid group was 0.91 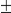 0.21 mg/dl	64.5
Shoji T 2000[33]	Biopsy-proven Ig A nephropathy	21	(1) Prednisolone 0.8 mg/kg/day, then reduced to 0.4 mg/kg/day 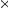 1 month and tapered to 10 mg 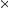 1 year(2) dipyridamole 300 mg/day 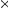 1 year	Proteinuria<1.5 g/day of protein and serum creatinine<1.5 mg/dl	12
K.N.Lai 1986[34]	Presence of IgA deposits in the mesangium	34	(1) Prednisolone 40–60 mg/day 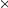 8 weeks, then tapered by half 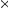 4 months(2) no corticosteroid therapy	Steroid group urinary protein was 6.5 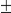 2.8 gm/day and non-steroid group was 4.7 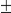 1.4 gm/day	38
Kobayashi Y 1996[35]	Fulfilled the three conditions defining the early stage of progressive Ig A nephropathy	90	(1) Prednisolone 40 mg/day 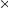 4 weeks, 30 mg/day 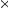 8 weeks, 25 mg/day 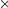 8 weeks, 20 mg/day 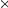 8 weeks, then 15 mg/day 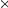 6 months and thereafter was further tapered(2) dipyridamole 300 mg/day	Creatinine clearance>70 ml/min	120
Welch TR 1992[36]	Been proved Ig A nephropathy	20	(1) Prednisolone 2 mg/kg(<80 mg) 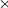 12 weeks(2) placebo	Serum creatinine<140umol/l	3
Kobayashi Y 1988[37]	Ig A nephropathy with primary glomerular deseases	96	(1) Prednisolone 40 mg/day 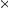 4 weeks, 30 mg/day 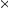 8 weeks, 25 mg/day 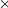 8 weeks, 20 mg/day 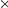 8 weeks, then 15 mg/day 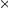 6 months and thereafter further tapered on a gradual basis(2) Non-steroidal anti-inflammatory and/or anti-thrombocyte drugs	Proteinuria>1.0 g/day	48
Julian BA 1993[38]	Biopsy-proven Ig A nephropathy	35	(1) Alternate day prednisolone 60 mg 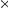 3 months(2) No treatment	Creatinine clearance>25 ml/min	24
Uzu T 2003[39]	Biopsy-proven Ig A nephropathy	45	(1) Methylprednisolone 1 g/day 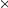 3 day, then oral prednisolone 40 mg/day 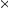 4 weeks, 30 mg/day 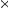 4 weeks, 25 mg/day 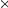 4 weeks, 20 mg/day 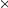 4 weeks, 15 mg/day 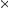 2 months, and gradual reduction in 6 months(2) Supportive treatment	Serum creatinine<106umol/l	36
Manno C[21]	Biopsy-proven Ig A nephropathy	97	(1) Prednisolone 1.0 mg/kg/day 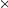 2 months, then 0.2 mg/kg/day 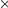 4 months(2) ramipril	Serum creatinine was 1.08 mg/dl	96
Lv J[22]	Biopsy-proven Ig A nephropathy	63	(1) oral prednisone 0.8 to 1.0 mg/kg/day 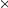 8 weeks, then the dose was tapered by 5 to 10 mg every 2 weeks(2) cilazapril	Serum creatinine was 1.1 mg/dl	48
Locatelli F 2001[40]	Biopsy-proven Ig A nephropathy	86	(1) Methylprednisolone 1 g/day 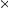 3 day at the beginning of vmonths 1, 3, and 5, and again two and four months later, oral prednisolone 0.5 mg/kg 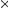 6 months(2) Supportive treatment	Plasma creatinine levels<1.5 ml/dL	64

**Table 2:** Risk of Bias in Studies.

Source	Intention-to-treat analysis	Allocation concealment	Blinding	Lost to follow up
Katafuchi R 2008 [28]	No	Unclear	Unclear	No lost to follow-up
Koike M 2008[29]	Yes	Unsealed envelops	Yes: patients and caregivers	No lost to follow-up
Pozzi C 2004[30]	Yes	Yes	Unclear	No lost to follow-up
Moriyama T 2004[31]	Yes	Unclear	No	No lost to follow-up
Katafuchi R 2003[32]	No	Yes	No	Lost to follow-up: 13 patients
Shoji T 2000[33]	No	Yes	No	Lost to follow-up:2 patients
K.N.Lai 1986[34]	No	Unclear	No	No lost to follow-up
Kobayashi Y 1996[35]	No	Inadequate	No	Lost to follow-up:44 patients
Welch TR 1992[36]	Unclear	Yes	Yes: patients and investigators	Lost to follow-up:3 patients
Kobayashi Y 1988[37]	No	Unclear	Unclear	No lost to follow-up
Julian BA 1993[38]	No	Yes	Unclear	Lost to follow-up:2 patients
Uzu T 2003[39]	No	No	Unclear	No lost to follow-up
Manno C[21]	Yes	Yes	No	Lost to follow-up: 6 patients
Lv J[22]	Yes	Yes	No	Lost to follow-up: 3 patients
Locatelli F 2001[40]	Yes	Unclear	Unclear	Lost to follow-up: 12 patients

### Effects of Interventions

**Figure 2:**
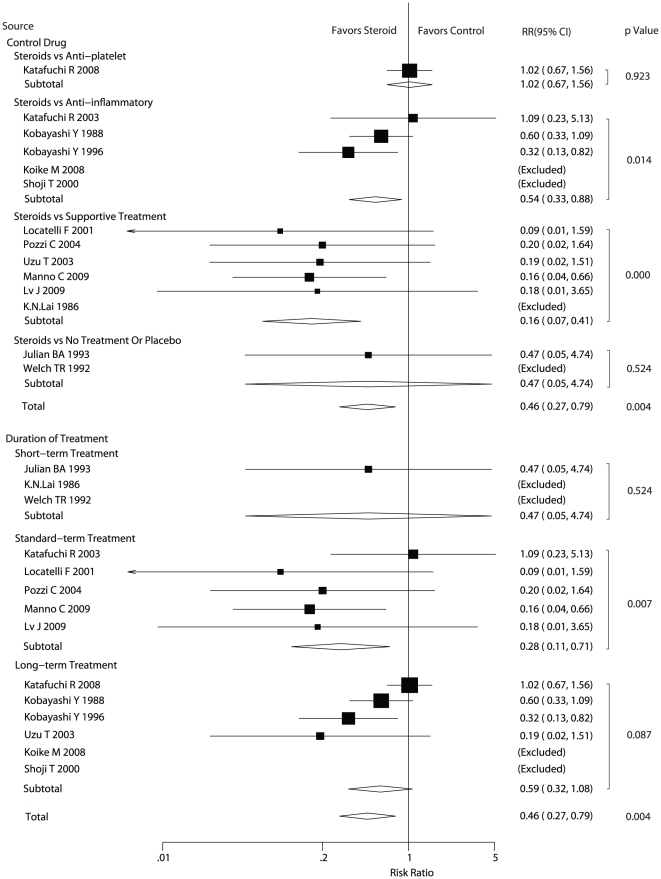
Effect of steroids on end-stage renal failure in patients with Ig A nephropathy. CI, confidence intervals; RR, relative risk.

Data for the effect of steroids on end stage renal failure were available from fourteen trials. We also performed subgroup analyses based on the type of control drug or the duration of steroid therapy. In Figure 2, the pooled RR showed that the application of steroid therapy had a statistically significant relationship with reduced the risk of end stage renal failure (RR: 0.46, 95%CI: 0.27 to 0.79). Subgroup analysis based on the type of control drug showed that compared with anti-platelet for the treatment of patients in control group, steroids was not associated with a statistically significant reduction of the risk in end stage renal failure. Furthermore, the study of steroid therapy vs no treatment or placebo also provided no evidence that steroid therapy could reduced the risk of end-stage renal failure. In contrast, steroid therapy reduced the risk of end-stage renal failure compared with anti-inflammatory (RR = 0.54, 95%CI: 0.33 to 0.88) or supportive treatment (RR = 0.16, 95%CI: 0.07 to 0.41) with a statistical significance. Subgroup analysis based on the duration of treatment indicated that standard term therapy could lead to lower risk of end stage renal failure when compared with control group (RR = 0.28, 95%CI: 0.11 to 0.71), while this could not be observed for short and long term steroid therapy.

**Figure 3:**
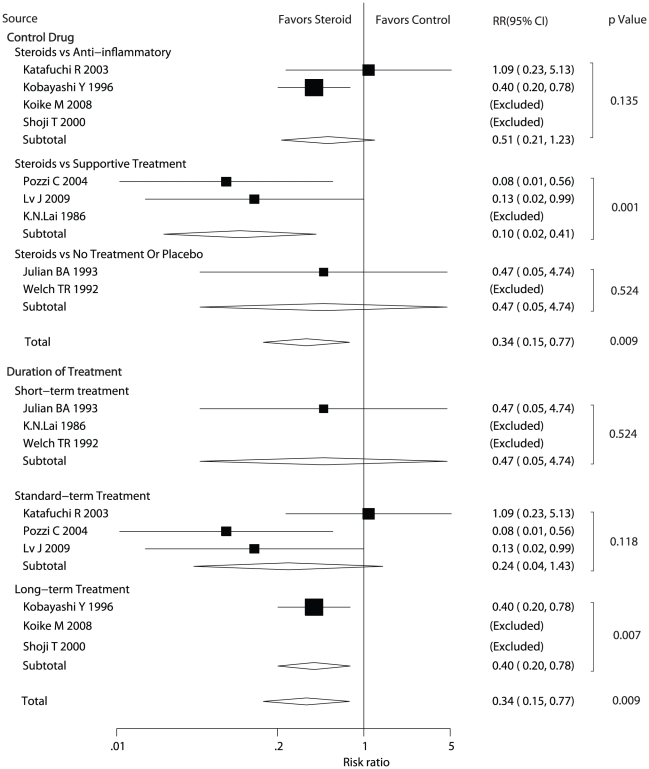
Effect of steroids on doubling of serum creatinine in patients with Ig A nephropathy. CI, confidence intervals; RR, relative risk.

Data for the effect of steroids on doubling of serum creatinine were available from nine trials. We also performed subgroup analyses based on the type of control drug or the duration of steroid therapy. As shown in Figure 3, steroid therapy was associated with a statistically significant reduction of the risk in doubling of serum creatinine (RR = 0.34, 95% CI: 0.15 to 0.77). Subgroup analysis based on the type of control drug suggested that steroid therapy was not associated with lower risk of doubling of serum creatinine when patients in control group received anti-inflammatory treatment, placebo or no treatment. In contrast, steroid therapy statistically reduced the risk of doubling of serum creatinine compared with supportive treatment (RR = 0.10, 95%CI = 0.02 to 0.41). In addition, subgroup analysis based upon the duration of treatment showed that compared with control group, long term steroid therapy could lead to lower risk of doubling of serum creatinine (RR = 0.40, 95%CI = 0.20 to 0.78), while this could not be observed for short and standard term steroid therapy.

**Figure 4:**
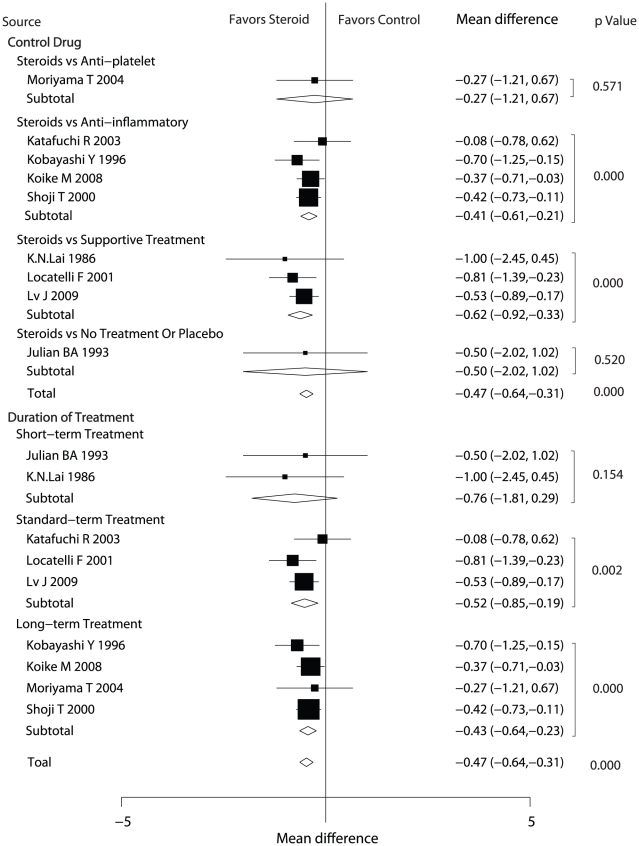
Effect of steroids on urinary protein excretion in patients with Ig A nephropathy. CI, confidence intervals; WMD, weighted mean difference.

Data for the effect of steroids on urinary protein excretion were available from nine trials (Figure 4). Statistical analyses showed that steroid therapy reduced urinary protein excretion compared with control therapy (MD = −0.47 g/day, 95%CI = −0.64 to −0.31) Subgroup analysis based on the type of control drug suggested that steroid therapy was not associated with reduced urinary protein excretion when patients in control group received anti-platelet treatment, placebo or no treatment. In contrast, steroid therapy led to statistically significant reduced urinary protein excretion compared with anti-inflammatory (MD = −0.41 g/day, 95%CI = −0.61 to −0.21) and supportive treatment (MD = −0.62 g/day, 95%CI = −0.92 to −0.33). Subgroup analysis based on the duration of treatment indicated that there was an association between significantly decreased urinary protein excretion and steroid therapy in standard term (MD = −0.52 g/day, 95%CI = −0.85 to −0.19) and in long term (MD = −0.43 g/day, 95%CI = −0.64 to −0.23). But for short-term treatment, there was no evidence showing that steroid therapy could reduce the urinary protein excretion.

In addition, the analysis of adverse effect events was difficult, because a majority of these studies reported few adverse effects. It is unclear whether there were no events or they were not properly recorded, and even worse some trials did not report adverse events intentionally. Furthermore, it should be noticed that the reported adverse events of treatment or control group are specific or nonspecific, such as stomach bleeding and unusual-hypertension [40], [41].

## Discussion

An increasing number of trials have evaluated the role of steroids in the treatment of IgA nephropathy and acquired various results, for which medical practitioners have few evidence-based treatment guidelines. In this large quantitative study, we performed a comprehensive literature search with no restrictions in publication status. More than 1542 patients were included to examine the effect of steroids on end-stage renal failure, doubling of serum creatinine and urinary protein excretion. The study suggested that compared with non-steroid therapy, steroid therapy was associated with clinically and statistically significant reduction of the risk in end stage renal failure (RR: 0.46, 95%CI: 0.27 to 0.79), doubling serum creatinine, (RR = 0.34, 95%CI = 0.15 to 0.77) and also urinary protein excretion at the end of treatment (MD = −0.47 g/day, 5%CI = −0.64 to −0.31).

Our results were consistent with the recommendations for the management of IgA nephropathy which were published in 1999 [13]. The recommendation indicated that IgA nephropathy patients should be treated with steroids to reduce proteinuria and stabilize renal function. However, the recommendation encountered criticism because it was based on a variety of sources, including non-randomized controlled trials data and with suboptimal methodological quality. Consequently, there was not enough information to provide strong evidence.

This meta-analysis showed that for patients with Ig A nephropathy, steroids could reduce the incidence of end stage renal failure, doubling serum creatinine and urinary protein excretion compared with patients receiving no steroidal treatment. Subgroup analysis showed that steroids vs anti-inflammatory could reduce the risk of end stage renal failure and the urinary protein excretion with a statistical significance. However, there was no evidence that steroid therapy protected against the doubling serum creatinine risk compared with anti-inflammatory therapy. The reason for this absence of difference could be that fewer events occurred and that the incidence of doubling serum creatinine was not reported in Kobayashi' study [37]. Furthermore, steroid therapy statistically reduced the risk of end-stage renal failure, doubling serum creatinine and also urinary protein excretion compared with supportive treatment. Subgroup analysis based upon the duration of treatment showed that compared with control group, long term steroid therapy was associated with a decrease of proteinuria and with a statistically significant reduction of the risk in end-stage renal failure, in addition, standard-term therapy was associated with a statistically significant reduction of the risk in end stage renal failure and urinary protein excretion. Finally, the *P* value for long-term therapy was very close to 0.05 for reducing the risk of end stage renal failure, which may be explained by the small amount of trials including results in this aspect. These findings corresponded with recent researches [40], [42], [43] that steroid therapy should be given for a long-term therapy.

To minimize the consequences of heterogeneity among the included studies, we performed a sequential exclusion of each trial from the pooled analysis. These exclusions did not affect the results of our meta-analysis.

Subgroup analysis is only done between-study hypothesis (not within-study hypothesis) in our review, because individual patient data and original data were not available. However, its validity was acceptable according to recently proposed criteria [44], [45], as we only conducted 2 subgroup analyses, which were based on the duration of treatment and on the type of control drug.

Recently, Cheng's review [46] illustrated that glucocoticoids significantly induced a protection of renal function and a reduction of proteinuria in patients with IgA nephropathy. This conclusion is in accordance with our own results. In addition, our subgroup analysis studied important factors which could affect the interpretation of our data. The results of this meta-analysis are promising because the outcomes consistently favor the use of steroids interventions. In all the interventions tested in the available trials, a long-term steroid therapy seems to be more beneficial.

The limitation of this study includes the inherent assumptions made for any meta-analysis, because the analysis uses pooled data either from published papers or provided by individual study authors, individual patient data and original data were not available, which prevented us doing more detailed relevant analysis and obtaining more comprehensive results. Furthermore, different follow-up times and therapy dose also could have affected our conclusions about the association between steroids and renal function. We also did not have sufficient data to get detailed effects of steroids on renal function. Therefore, we just gave a relative result by comparing steroid therapy with non-steroid therapy and provided a synthetic and comprehensive review.

For future research, promising interventions should be tested, including dosage, duration of treatment or combination with other influencing factors, such as cyclophosphamide, fish-oil, dipyridamole, antiplatelet drugs, etc, through which we might confirm the optimal time of treatment, the optimal dosage and the optimal therapy [47]. We suggest that ongoing trials would be improved by the following ways: i). Adverse events of clinical trials should be recorded and reported ii). Treatment duration and dosage should be taken into consideration before evaluating clinical outcomes.
